# Eugenol eliminates carbapenem-resistant *Klebsiella pneumoniae* via reactive oxygen species mechanism

**DOI:** 10.3389/fmicb.2023.1090787

**Published:** 2023-02-16

**Authors:** Wei Liu, Guang Chen, Keke Dou, Bingcheng Yi, Danyang Wang, Qihui Zhou, Yunbo Sun

**Affiliations:** ^1^Department of Critical Care Medicine, College of Medicine, The Affiliated Hospital of Qingdao University, Qingdao University, Qingdao, China; ^2^Department of Stomatology, The Affiliated Hospital of Qingdao University, Qingdao University, Institute for Translational Medicine, Qingdao, China; ^3^School of Rehabilitation Sciences and Engineering, University of Health and Rehabilitation Sciences, Qingdao, China

**Keywords:** eugenol, carbapenem-resistant *Klebsiella pneumoniae*, reactive oxygen species, antibacteria, biofilm

## Abstract

Multidrug-resistant (MDR) bacterial infections have gained increasing attention due to the high incidence rates and high mortality, especially for the carbapenem-resistant *Klebsiella pneumoniae* (CRKP) infection that can cause severe complications (e.g., pneumonia and sepsis) in multiple organs. Therefore, the development of new antibacterial agents against CRKP is imperative. Inspired by natural plant antibacterial agents with broad-spectrum antibacterial properties, the antibacterial/biofilm activity of eugenol (EG) on CRKP and their underlying mechanisms are investigated in our work. It is found that EG exhibits remarkable inhibitory effects on planktonic CRKP in a dose-dependent fashion. Meanwhile, the destruction of membrane integrity induced by the formation of reactive oxygen species (ROS) and glutathione reduction results in the leakage of bacterial cytoplasmic components, including DNA, β-galactosidase, and protein. Moreover, when EG contacts with bacterial biofilm, the whole thickness of the dense biofilm matrix decreases, and the integrity is destroyed. Overall, this work verified that EG could eliminate CRKP *via* ROS-induced membrane rupture, which offers vital evidence to explain the antibacterial ability of EG against CRKP.

## 1. Introduction

Multidrug-resistant bacteria (MDR) infections, featuring high incidence and fatality rates, are a threat to public health (Liu et al., 2020; Yu et al., 2020; Mei et al., 2021). Among them, carbapenem-resistant *Klebsiella pneumoniae* (CRKP) has been listed as one of the most critical drug-resistant pathogens by the World Health Organization, because its carbapenemases product, capable of hydrolyzing and inactivating carbapenems and β-lactams antibiotics, can result in the dramatical infection occlusion in bloodstream, urinary tract, surgical-site, and lung (Broberg et al., 2014; Escolà-Vergé et al., 2020; Mehrbakhsh et al., 2020; Han et al., 2022; Liu et al., 2022). To treat the bacterial infections, extensive efforts have been devoted and remarkable progresses have been made using various antibiotics (Ji et al., 2021; Zong et al., 2022). Nevertheless, the removal effect of CRKP remains far from ideal due to the special natures of the bacteria (e.g., carbapenemases products, biofilm formation, and high mutation rate) that help CRKP to escape the clearance by the host immune system and most of the available antimicrobial agents (Di Domenico et al., 2020). In this context, developing new therapeutic and reliable alternatives for eradicating CRKP pathogens are urgently required to combat the imminent deadly infections.

Considering the current developments of antimicrobial strategies, natural plant antibacterial agents with broad-spectrum antimicrobial properties have attracted considerable attention and have been applied in this field (Salam and Quave, 2018; Doyle and Stephens, 2019; Salem et al., 2021). Eugenol (EG), as bioactive extract-of clove oil, has been proven to exhibit various pharmacological activities, including anti-inflammatory, antioxidant, analgesic, and anesthetic (Bezerra et al., 2017; Mohammadi Nejad et al., 2017; Barboza et al., 2018; Hui et al., 2020; Putland et al., 2020). Meanwhile, it also received increasing concern as a new antimicrobial recently (Bezerra et al., 2017; Marchese et al., 2017; Zari et al., 2021). For example, EG was demonstrated to be capable of interfering with the biofilm formation of *Vibrio parahaemolyticus* and *Candida albicans* attributing to its double bonds at the α, β positions of the side chain and a methyl group at the γ position, which thereby inhibited the subsequent biofilm-related infections (Khan and Ahmad, 2012; Nazzaro et al., 2013; De Paula et al., 2014; Ashrafudoulla et al., 2020). This special mechanism indicated the broad-spectrum antibacterial effect of EG on gram-negative bacteria, gram-positive bacteria, as well as drug-resistant bacteria. As such, EG was also observed to present excellent antimicrobial activity against CRKP growth and its biofilm formation recently (Qian et al., 2020b). Despite this, the detailed molecular mechanisms are still elusive regarding EG-modulated CRKP infections. Interestingly, Das et al. verified that EG could effectively kill vancomycin-resistant *S. aureus* strains and *Shigella flexneri via* inducing the generation of reactive oxygen species (ROS) and destroying bacteria membranes (Das et al., 2016; Bai et al., 2022). In addition, eugenol can lead to oxidative damage of cell membrane through the increase of ROS level in cells, destroy the permeability and integrity of cell membrane, and then lead to the death of *Candida albicans* and inhibition of biofilm (Shahina et al., 2022). Therefore, understanding whether the ROS mechanism mediate the EG-eliminated CRKP infections is fundamental for advancing the clinical application of EG.

Herein, the inhibitory effects of EG on bacteria growth and biofilm formation of CRKP were analyzed firstly using the CFU count test and LIVE/DEAD bacterial viability kit. Then, the bacterial morphology and membrane permeability of CRKP was further observed *via* scanning electron microscopy (SEM), transmission electron microscopy (TEM), and intracellular leakage test, in order to confirm the antibacterial activity of EG against CRKP. Lastly, the ROS generation of CRKP was detected using 2,7-dichlorodihy-drofluorescein diacetate dye (DCFH-DA) and the relevant antibacterial mechanism of EG in causing CRKP death was discussed.

## 2. Materials and methods

### 2.1. Materials

Eugenol (purity: 98.5%) was provided by Solarbio Science & Technology Co., Ltd. (Beijing, China). Tryptic Soy Broth (TSB), agar, and 2,7-dichlorodihy-drofluorescein diacetate dye (DCFH-DA) were bought from Solarbio Science & Technology Co., Ltd. (Beijing, China). O-Nitrobenzene β-D-galactoside (ONPG) and bicinchoninic acid (BCA) Protein Assay Kit were purchased from Aladdin Bio-Chem Technology Co., Ltd. (Shanghai, China). The LIVE/DEAD^™^ BacLight^™^ bacterial viability kit was obtained from Thermo Fisher Scientific (United Kingdom). Thiobarbituric acid (TBA), trichloroacetic acid (TCA), and 5–5′-dithiobis (2-nitrobenzoic acid; DTNB) were purchased from Aladdin Bio-Chem Technology Co., Ltd. (Shanghai, China). Without further purification, all reagents were used directly. Double distilled water (DDW) was used in the experiment.

### 2.2. Antibacterial assay

#### 2.2.1. Carbapenem-resistant *Klebsiella pneumoniae* culture

The clinical strain of CRKP separated and identified by the Department of Critical Care Medicine and the Laboratory Department of the Affiliated Hospital of Qingdao University was used in this study. On a TSB agar plate, the bacteria were activated and quadrant-streaked. Single CRKP colonies were cultured in 8 ml of TSB medium overnight at 37°C with overnight shaking. Following the dilution of the bacterial suspension with TSB media, the OD600 value for the fixed suspension was determined at 0.03–0.05, to maintain the number of bacteria in 10^5^ ~ 10^7^ (Tang et al., 2022).

#### 2.2.2. Anti-CRKP test

Eugenol (EG) with final concentrations of 0.25, 0.5, and 1.0 mg/ml was added into bacterial suspensions at 37°C for 24 h, respectively. TSB medium and DMSO (1%) were co-cultured with bacteria as control groups. For each sample, diluted suspensions were prepared by incubating them for 24 h at 37°C. Then the volume of 20 μl bacterial diluent was dropped on the TSB agar plate to make it evenly distributed, and cultured at 37°C for 24 h. Each group was tested three times. The inhibition ratio was calculated as follows:


(1) $$\mathrm{Inhibition ratio}\;\left(\%\right)=\left(1-\frac{\mathrm{CFU}\;\left(\mathrm{each group}\right)}{\mathrm{CFU}\;\left(\mathrm{control}\right)}\right)\times 100\%.$$

#### 2.2.3. Growth curves of bacteria

In a 96-well plate, 100 μl of EG with final concentrations of 0.25, 0.5, and 1.0 mg/ml and 100 μl of CRKP (2 × 10^3^ CFU/ml) were simultaneously added to each well and mix evenly. TSB medium and DMSO (1%) were co-cultured with bacteria as control groups. Measurements of OD600 by BioTek enzyme-labeled instrument (America) every hour for 24 h were used to determine the growth-inhibitory curve for bacteria. The experiment was repeated three times independently.

#### 2.2.4. Anti-biofilm evaluation

Placed the 14 mm diameter glass coverslips into a 24-well plate after sterilizing with 75% alcohol, the 500 μl suspension of CRKP (OD600 = 0.05) was dripped into a 24-well plate and incubated under a constant environment for 12 h to form biofilms for the subsequent experiments. The CRKP biofilms were co-cultured with blank, DMSO (1%), 0.25, 0.5, and 1.0 mg/ml EG for 36 h. Following the removal of excess bacteria with PBS, biofilms were stained for 20 min with a LIVE/DEAD^™^ BacLight^™^ bacterial viability kit. Using the confocal laser scanning microscope (CLSM, Leica TCS SP8, Germany), we visualized the fluorescence of the biofilms after washing them with PBS. Imagers used a single channel and a dual channel to image green and red fluorescence. LAS AF software was used for the acquisition of CLSM images of biofilms (version 2.6.0.7266, Leica Microsystems CMS GmbH). COMSTAT 2.1 was used to quantify Live/Dead bacteria. Three independent experiments were conducted.

### 2.3. Membrane structure and permeability of CRKP

#### 2.3.1. Scanning electron microscopy

Bacteria were co-cultured with EG in different concentrations at 37°C for 24 h, TSB medium and DMSO (1%) were co-cultured with bacteria as control groups, centrifuged for 5 min at 5000 rpm, and then washed three times with PBS. To dehydrate bacterial clumps, 2.5% glutaraldehyde at 4°C was used for 4 h, followed by graded 30, 50, 70, 90, and 100% alcohol. After fixing the samples on SEM support and coating them with gold under vacuum, the morphology of bacteria was observed using SEM (VEGA3, TESCAN, Czech).

#### 2.3.2. Transmission electron microscopy

Through TEM (Mic JEM-1200EX, Japan), we observed the morphology and integrity of bacteria. In brief, we co-cultured the blank, DMSO (1%), EG 0.25, EG 0.5, and EG 1.0 groups with bacteria suspensions for 24 h. Afterward, they were centrifuged at 5000 rpm for 5 min, rinsed three times with PBS, and fixed with glutaraldehyde at 2.5% (*v*/*v*). TEM was then used to observe the bacterial membrane of CRKP.

#### 2.3.3. Membrane permeability of bacteria

As a membrane-impermeable chromogenic substrate, ONPG cannot pass through the inner membrane of bacteria unless the inside membrane is damaged (Sun et al., 2021). Therefore, ONPG was used to evaluate the bacteria’s membrane permeability. As mentioned in the section on antibacterial tests of EG, 15 μl of bacterial suspension was collected from each group. Following that, 110 μl PBS, 15 μl ONPG solution, and 10 μl DMSO (1%) were mixed into the above bacterial mixture in a 96-well plate. After 30 min of treatment, 1 M Na_2_CO_3_ was added to stop the reaction. Lastly, ONPG can be determined from the absorbance of the bacteria suspension at 420 nm. The experiment was repeated three times independently.

### 2.4. Mechanism analysis and verification

#### 2.4.1. Evaluation of intracellular ROS formation

2,7-Dichlorodihy-drofluorescein diacetate dye (DCFH-DA) was used as an intracellular ROS probe to detect ROS production inside bacteria. As with the antibacterial experiment, shake each suspension of co-cultured bacteria thoroughly after 24 h. By centrifugation, the supernatants were removed after the suspensions being centrifuged for 5 min at 5000 rpm. CRKP and DCFH-DA solutions were mixed at the bottom of the test tubes according to the instructions at 37°C for 20 min. To measure fluorescence intensity, a microplate reader was used. The excitation wavelength is 488 nm, and the emission wavelength is 525 nm. The experiment was repeated three times independently.

#### 2.4.2. Detection of nucleic acid leakage and protein

The cytoplasmic components of the bacteria are released once the membrane of the bacteria is destroyed. A 260 nm absorbance measurement can indicate how much nucleic acid is released by bacteria. As mentioned in the part of antibacterial tests of EG before centrifugation of bacterial suspensions, the supernatant was obtained and the OD260 was determined by NanoDrop One. In addition, Protein loss would occur when membrane permeability changes. Protein leakage is another indicator of membrane permeability change. A 96-well plate was filled with 100 μl of bacterial suspensions from each group, and the protein concentration leaked was monitored and quantified by a BCA protein detection kit. The experiment was repeated three times independently.

#### 2.4.3. Thiobarbituric acid assay and reduced glutathione (GSH) assay

ROS destroying the bacterial membrane generally accompanies with the production of lipid peroxide radical, thereby forming the malondialdehyde (MDA). To verify the role of ROS in breaking the bacterial membrane (Ayaz Ahmed and Anbazhagan, 2017), MDA expression was detected by TBA assay. Briefly, 10% TCA was introduced into the bacterial liquid that was co-cultured with EG for 24 h, followed by the addition of 0.67% TBA to incubate for 1 h at 95°C. After being cooled to room temperature, the reaction mixture was centrifuged at 6000 rpm for 15 min, and the absorbance of the supernatant was measured at 532 nm using a microplate reader. Bacteria treated with 10 μM hydrogen peroxide and untreated bacteria were used as positive and negative controls, respectively.

In addition, excessive ROS is inclined to reduce the intracellular concentration of GSH and then weaken the anti-oxidation ability of bacteria, thus leading to lipid peroxidation of the membrane (Rahman et al., 2007). Hence, GSH expression induced by EG was detected by DTNB assay. Briefly, the bacterial liquid, co-cultured with EG for 24 h, was collected and cracked on ice with 10% TCA solution for 15 min. Then, 200 μl of the bacterial lysate was mixed with 1,800 μl Tris buffer (30 mM, pH 8.3) and 100 μl 0.1% DTNB for the incubation of 90 min in the dark at room temperature. After that, the absorbance of solutions was monitored at 412 nm using a microplate reader. Bacteria treated with 10 μM hydrogen peroxide and untreated bacteria were used as positive and negative controls, respectively. The relative production of MDA or GSH was estimated as the following formula:


(2) $$\begin{array}{l} \mathrm{Relative production of}\;\mathrm{MDA}\;\mathrm{or}\;\mathrm{GSH}\;\left(\%\right)\\ =\frac{\left(ODs-ODn\right)}{\left(ODp-ODn\right)}\times 100\% \end{array}$$

Where *OD*_*s*_, *OD*_*n*_, and *OD*_*p*_ represent the detected absorbance of solutions for the sample group, negative and positive control, respectively.

### 2.5. Statistical analysis

All data points were presented as mean values ± standard deviation (SD). In order to analyze the differences between the groups, a one-way analysis of variance with Tukey’s test was carried out using GraphPad Prism 8.0. Different numbers of asterisks indicate the significance differences, *p < 0.05, **p < 0.01, and ***p < 0.001.

## 3. Results and discussion

### 3.1. Eugenol exhibited excellent antibacterial/biofilm effect against CRKP

**Figure 1:**
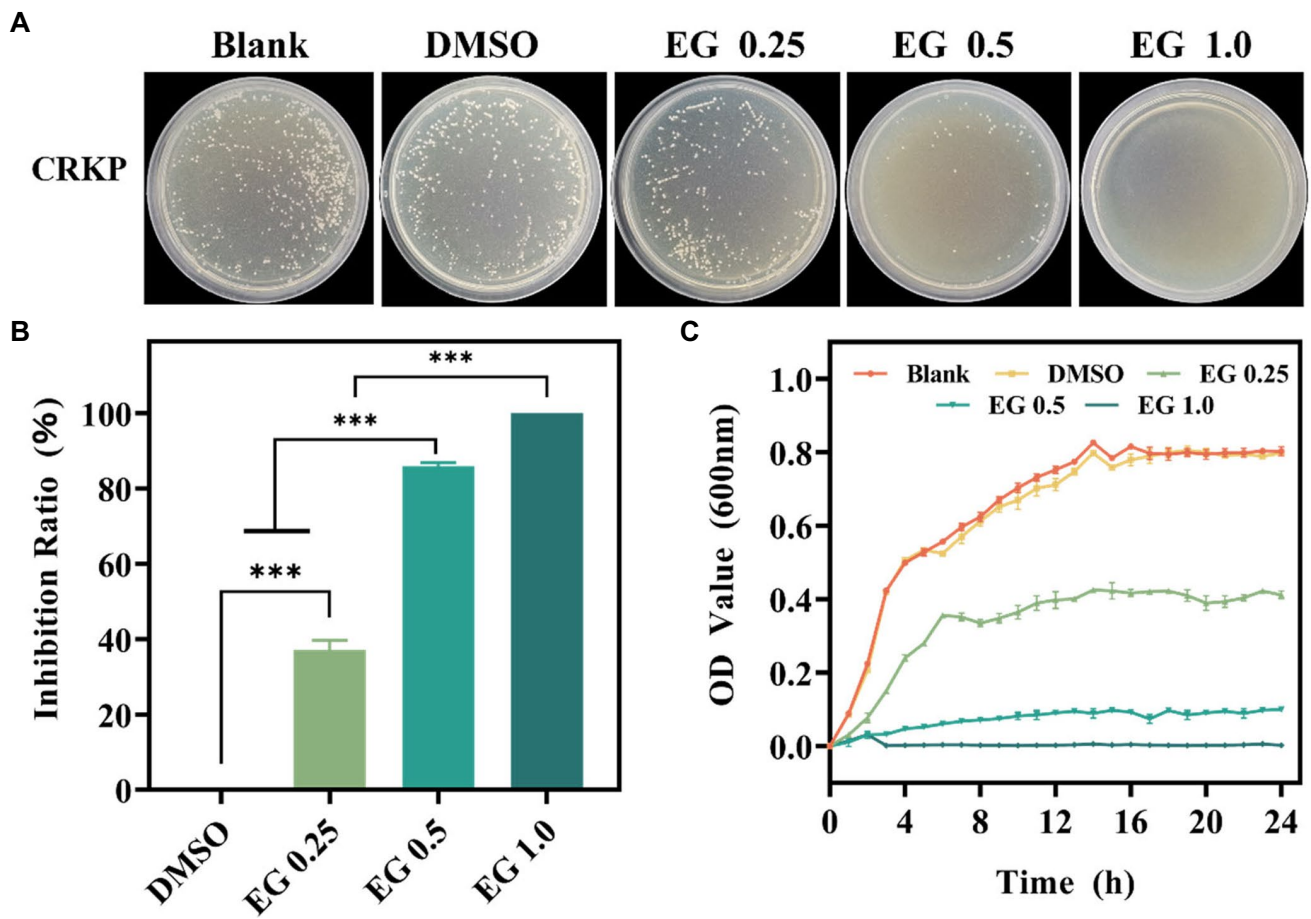
**(A)** The colony photographs of carbapenem-resistant *Klebsiella pneumoniae* (CRKP) on agar plates after treatment with eugenol (EG) of corresponding concentrations. **(B)** The calculated inhibition ratio of CRKP at corresponding concentrations of EG. **(C)** Growth curves of CRKP during co-cultured with EG. ns: *p* > 0.05, ^***^p < 0.001. All data are presented as mean ± SD (*n* = 3).

The antibacterial effect of EG against CRKP was assessed using the CFU count test. As shown in Figure 1A, there is a qualitatively reduced number of bacteria on the EG compared to the blank group. DMSO (1%) and blank groups showed no significant differences. It shows that DMSO (1%) did not affect the growth of bacteria (De Almeida et al., 2018; Kwiatkowski et al., 2022). Importantly, the number of bacteria in colonies gradually decreased as the concentration of EG increased from 0.25 to 1.0 mg/ml. The inhibition ratio of bacteria was calculated to better understand the antimicrobial effect of EG. Figure 1B shows that more than 85% of CRKP were killed by 0.5 mg/ml of EG, demonstrating the excellent antibacterial activity of EG. Furthermore, when the concentration of EG was 1.0 mg/ml, almost 100% of CRKP was eradicated. The effect of EG on the growth curve of CRKP was further detected in each dose. As depicted in Figure 1C, in the first 14 h, the concentration of CRKP in each group increased rapidly, reaching the exponential growth phase, and then entered a stable growth period in the following 10 h. Besides, EG showed a better inhibition effect than the group of blank and DMSO (1%) during 24 h. The results showed that EG significantly inhibited the growth of CRKP in a concentration-dependent manner.

**Figure 2:**
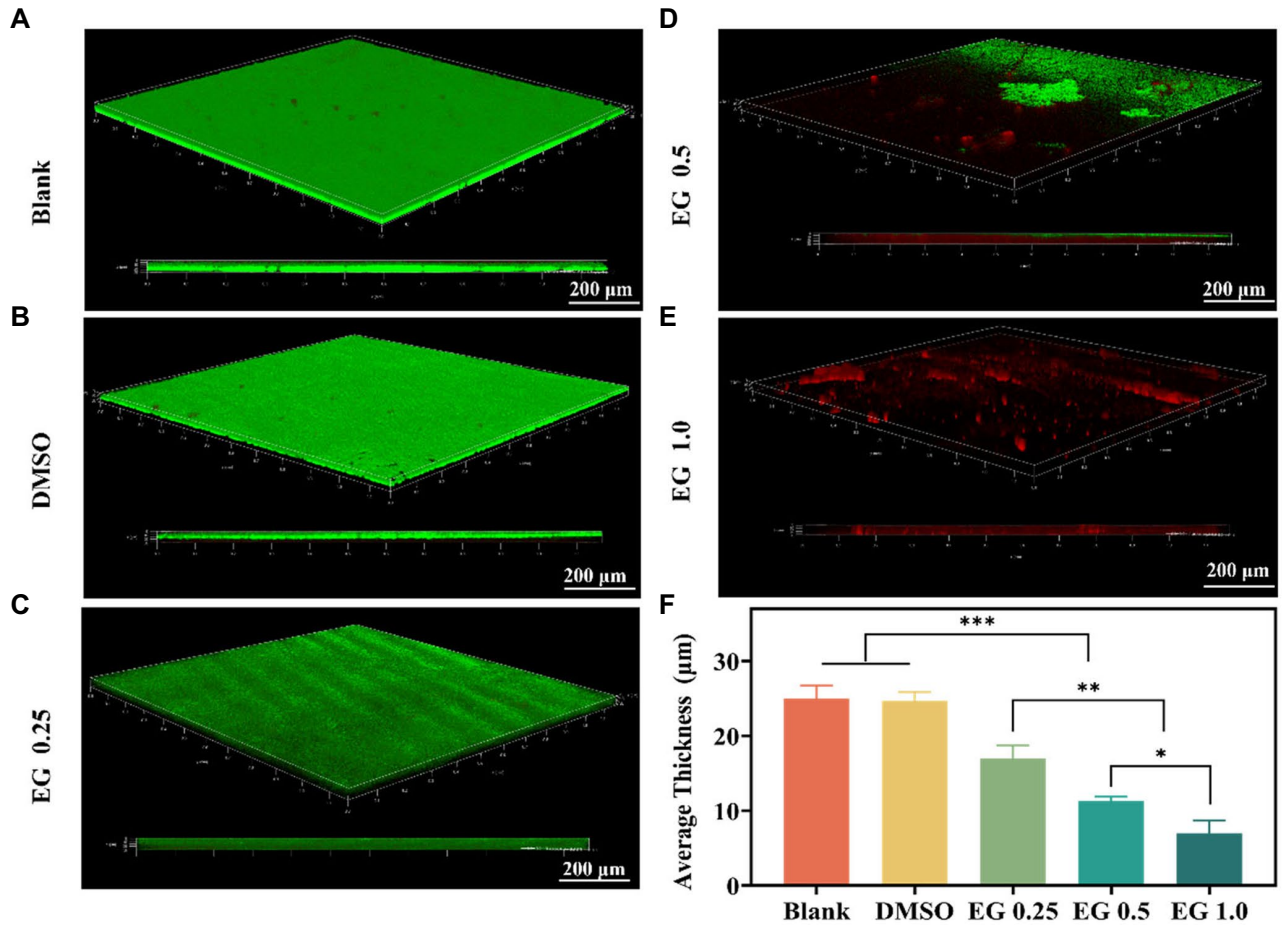
confocal laser scanning microscope (CLSM) images of CRKP biofilms were grown for 12 h and treated with EG for 36 h. Biofilms grown in **(A)** Blank, **(B)** DMSO (1%), **(C)** EG 0.25, **(D)** EG 0.5, **(E)** EG 1.0. Green is live bacteria. Red is dead bacteria. Scale bars = 200 μm. **(F)**The average thickness of CRKP biofilms. Data are mean ± SD (*n* = 3), ^*^p < 0.05, ^**^p < 0.01, ^***^p < 0.001.

Biofilms act as a barrier that protects bacteria from phagocytosis, which contributes to the limitations of the therapeutic options against CRKP (Carbapenem et al., 2019; Papayannopoulos, 2019; Tian et al., 2021). To further verify the antibiofilm properties of EG, we conducted antibacterial experiments on the biofilm of CRKP. Because of the strong drug resistance of biofilm, the anti-biofilm experiment, requiring a longer co-culture time than other antibacterial experiments, was performed after 36 h of culture (Qian et al., 2020a). CRKP biofilm was treated with different concentrations of EG for 36 h after being co-cultured at 37°C for 12 h. CLSM was used to observe the CRKP biofilms stained with a LIVE/DEAD^™^ fluorescence kit. As shown in Figure 2A,B, blank and DMSO (1%) coverslips were found to be completely covered in CRKP biofilm. On the contrary, after EG treatment, the surface coverage and thickness of the CRKP biofilm decreased (Figures 2C-E), confirming that the bacteria in the biofilm were destroyed. Based on the quantitative result in Figure 2F, the average thickness of biofilm treated with EG decreased significantly, and the effect of anti-biofilm was concentration-dependent when compared with the blank group. These results indicate that EG can inhibit and destroy CRKP biofilm.

### 3.2. Eugenol induced bacterial death by damaging the cell membrane

**Figure 3:**
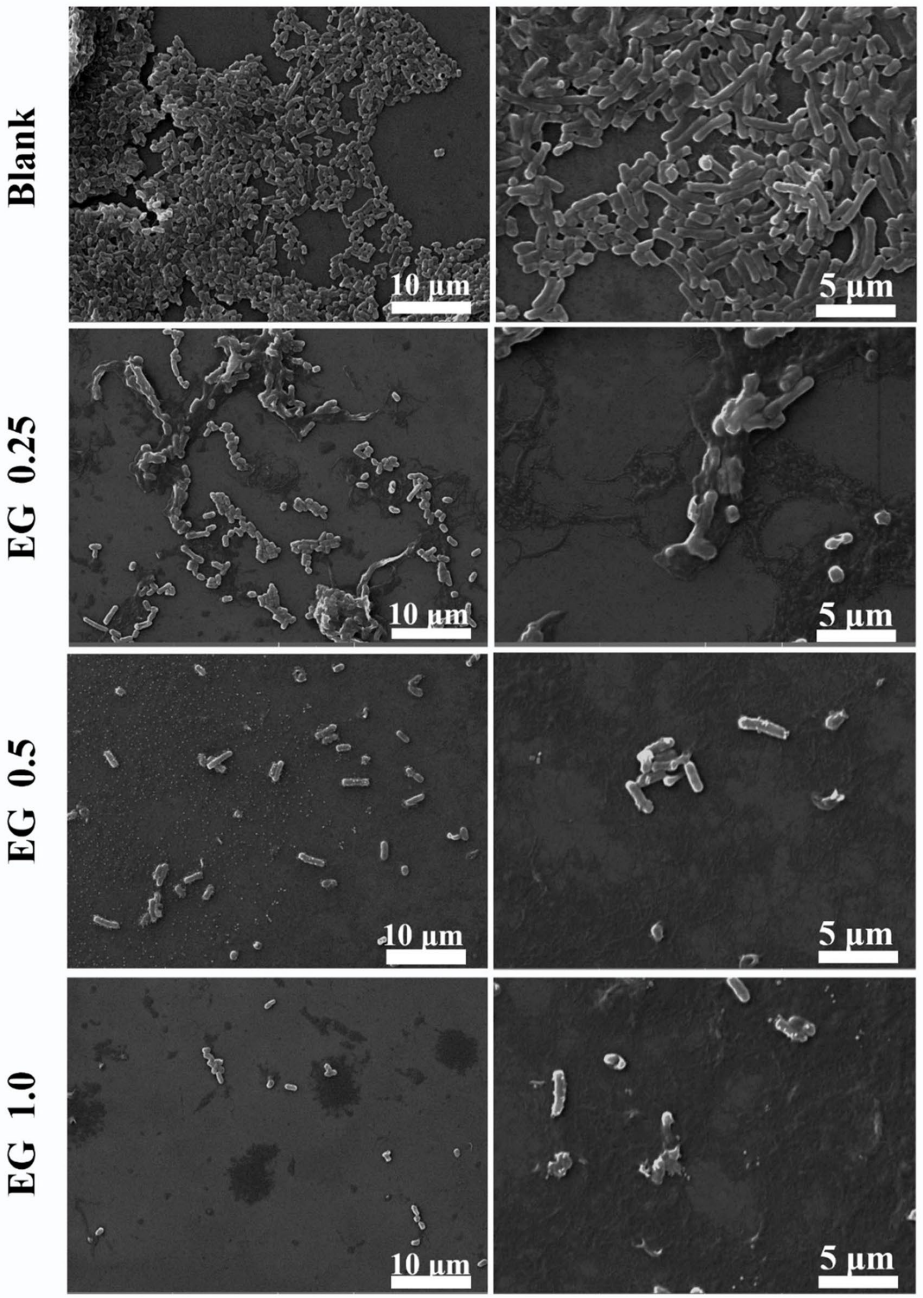
Morphology of CRKP bacteria under scanning electron microscopy (SEM) (5000 × and 10000 ×): blank and EG-treated CRKP strains.

In order to observe the change in bacterial morphology of CRKP after EG treatment, the bacteria were co-cultured with different concentrations of EG at 37°C for 24 h and then centrifuged for dehydration. The structure and morphology of bacteria before and after EG treatment were observed by SEM (Figure 3). The surface of the normal bacteria was smooth, intact, and showed typical characteristics, while the treated bacteria with EG were damaged severely. Some bacteria showed leaks, misshapen structures, and fragmented membranes.

**Figure 4:**
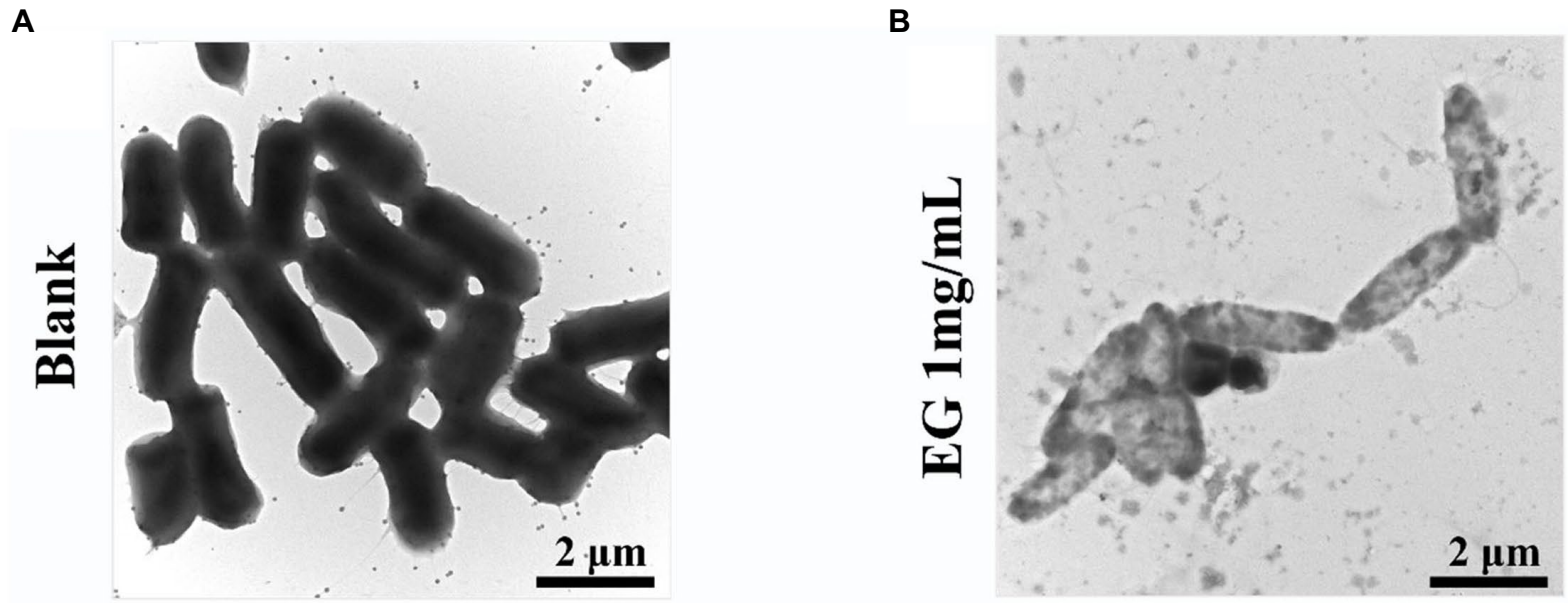
The structural integrity of bacteria membrane under TEM (15000×): **(A)** blank; **(B)** EG-treated CRKP strains.

To show the changes in bacterial cell integrity and internal material density of CRKP after EG treatment, we selected TEM for observation. TEM images display that bacteria membrane damage was observed in EG treated group. The density of cytoplasm was much lower than blank, which might be caused by the loss of cytoplasmic solute. Overall, the results of TEM were consistent with those of SEM, which further confirmed that EG exhibited resistance to CPKP by destroying the bacterial membrane (Figure 4).

### 3.3. Eugenol promoted ROS formation and intracellular substance efflux

**Figure 5:**
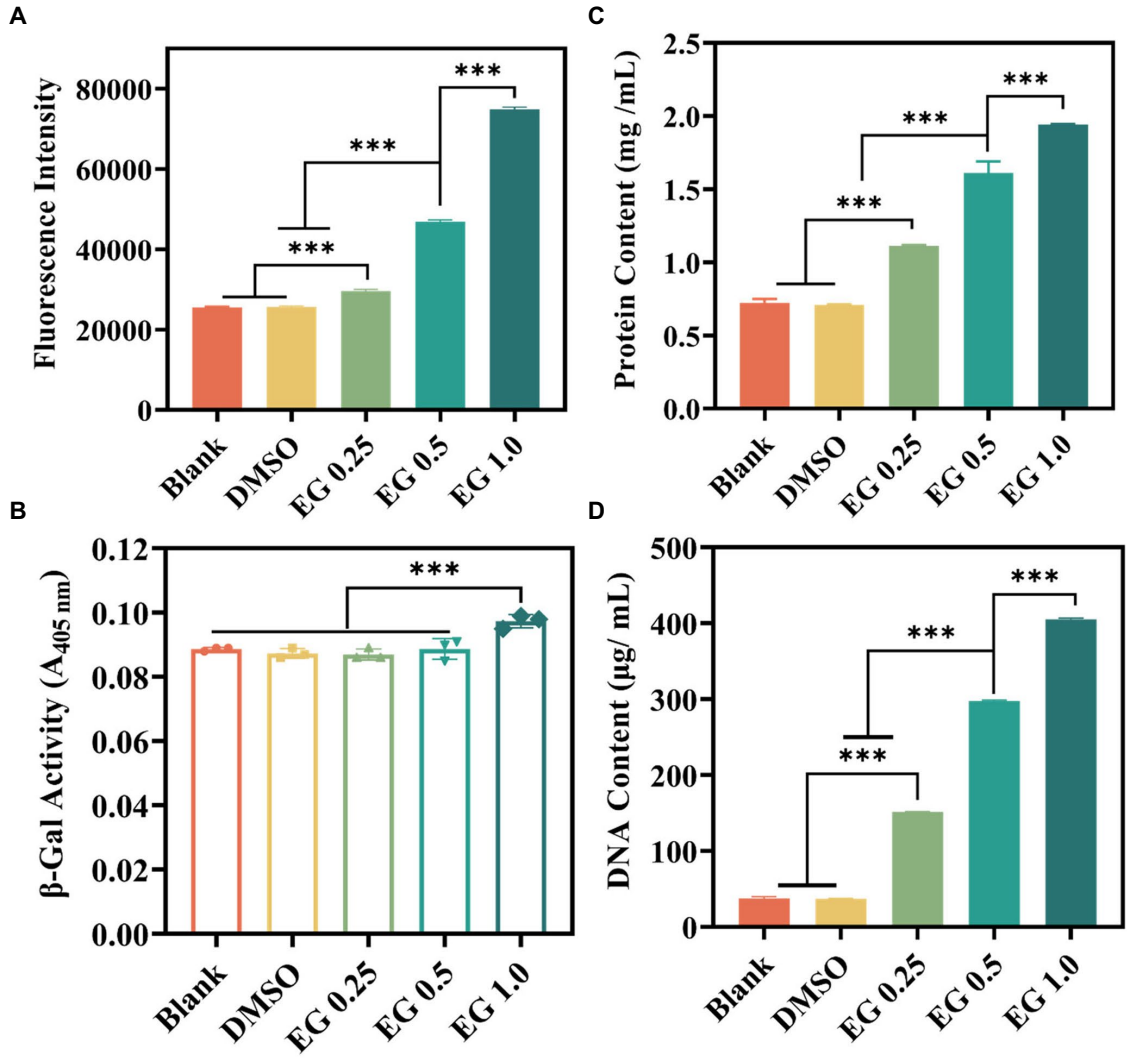
**(A)** Intracellular reactive oxygen species (ROS) generation of CRKP after treatment with EG for 24 h. **(B)** β-galactosidase leakage from the destroyed bacterial membrane by monitoring the value of A405 nm. **(C)** Total leaky proteins assessment of bacteria in the different groups. **(D)** DNA leakage content of the diverse groups. Data are mean ± SD (*n* = 3; ^***^p < 0.001).

Excessive active oxygen will cause lipid peroxidation, reduce the fluidity of the cell membrane, change the characteristics of the cell membrane, and then destroy the protein of the cell membrane, leading to bacterial death (Ezraty et al., 2017; Qi et al., 2020). To verify whether the above inhibitory activity of bacteria was associated with the release of ROS, ROS levels were measured using a ROS assay kit (Fan et al., 2021; Li et al., 2021; Lin et al., 2021). DCFH-DA was used to determine the EG-induced intracellular ROS generation in CRKP. As indicated in Figure 5A, after 24 h of co-cultured between EG and CRKP, the intracellular fluorescence intensity of bacteria was higher than that of the blank and DMSO (1%) group, and significantly enhanced with the increase of EG concentration. It was found that EG induced the high expression of ROS in cells to resist bacteria, which may be the mechanism of EG resistance to CRKP. Furthermore, it could cause an increase in oxidative stress and bacterial permeability, destroy the bacterial wall, and leak fluids within the bacteria. To confirm it, we analyzed cytoplasmic membrane permeability by collecting the supernatants from CRKP suspensions. It was noted that when the concentration of EG reached 1.0 mg/ml, the hydrolysis degree of ONPG and the cytoplasmic permeability were markedly increased (Figure 5B). Subsequently, we measured the DNA and protein concentrations in different groups of suspensions (Figure 5C/D). Compared to the blank and DMSO (1%), the concentration of DNA and protein was significantly higher in the EG group, suggesting that the membrane damage caused by EG led to the release of protein and DNA. Taken together, EG could compromise bacteria structure and induce the loss of intracellular components of CRKP.

**Figure 6:**
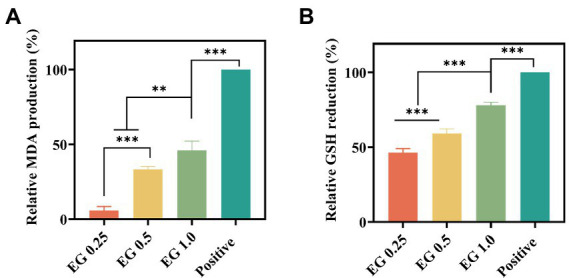
Effect of EG on the malondialdehyde (MDA) expression **(A)** and intracellular GSH expression **(B)** in the bacteria. ^**^p < 0.01, ^***^p < 0.001.

Considering that the unsaturated fatty acids of cell membranes are highly vulnerable to ROS attack, MDA production was measured to verify the mechanism that EG-induced ROS breaks the bacterial membrane *via* lipid peroxidation. As shown in Figure 6A, the MDA production in bacteria was positively associated with EG concentration, which supports the occurrence of lipid peroxidation induced by ROS in the bacterial membrane. GSH, a non-protein tripeptide that maintains the redox environment, can protect cells from oxidative damage induced by ROS. In turn, the excessive ROS oxidizes GSH to form disulfide (Vecitis et al., 2010). Thus, to directly verify the oxidative damage of EG-induced ROS to bacteria, the GSH amount was determined by DTNB assay. Results showed that EG significantly decreased the GSH concentration in the bacteria (Figure 6B). It indicated that EG could promote ROS expression and inhibit bacterial growth by reducing GSH. Overall, this evidence suggests that EG executes the antibacterial activity against CRKP *via* inducing the high ROS expression and GSH reduction, which then breaks the bacterial membrane.

**Figure 7:**
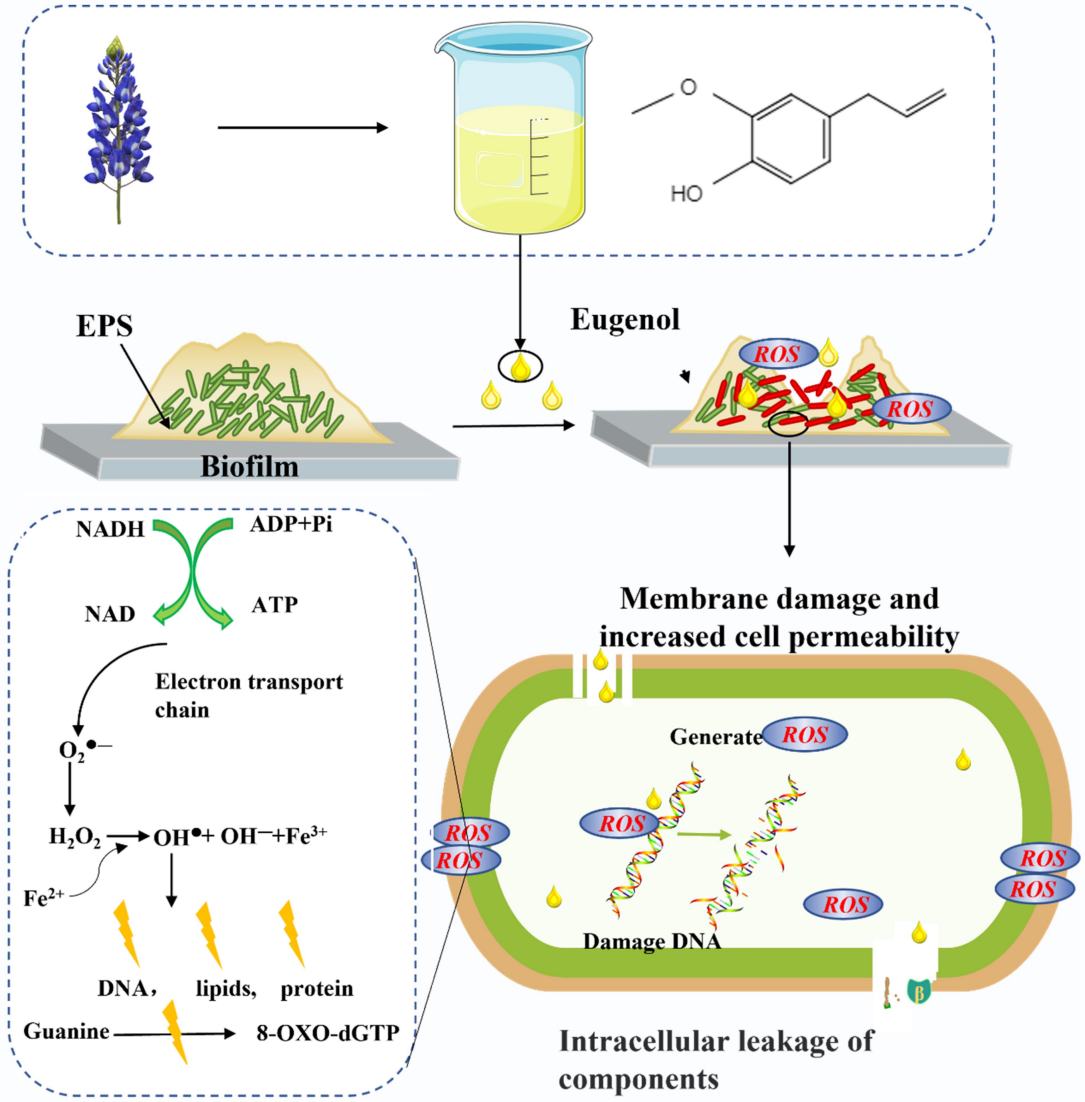
Schematic diagram of antibacterial mechanism of EG.

Most ROS in bacteria is produced in cell membranes by continuous univalent electron transfer reactions of oxygen molecules catalyzed by NAD(P)H oxidase in the respiratory chain (Dharmaraja, 2017; Van Acker and Coenye, 2017). Previous studies demonstrated that DNA, RNA, lipids, and protein in organisms were sensitive to ROS, and excessive ROS could cause damage to these substances (Borisov et al., 2021). The lipid peroxidation would change the characteristics of cell membrane, and then destroy the cell membrane proteins, leading to bacterial death (Ezraty et al., 2017). In addition, ROS could also destroy the double-stranded structure of DNA, affect the normal replication of DNA, and increase the probability of mutation (Srinivas et al., 2019). Therefore, in this context, the increased expression of ROS induced by EG has capable of changing the morphology of bacterial membrane through lipid peroxidation of cell membrane, which then increases the permeability of the bacterial membrane and leads to the leakage of cell substances, DNA damage, and even bacterial death (Srinivas et al., 2019). This shows that EG causes lipid peroxidation of the cell membrane to destroy the cell membrane by mediating ROS production, which directly affects the leakage of biological molecules in bacteria, further leading to bacterial death. The detailed antibacterial mechanism of EG against CRKP was shown in Figure 7.

## 4. Conclusion

In summary, EG showed efficient antibacterial properties against CRKP. Moreover, the antibacterial activity of EG was greatly dependent on their concentrations. When its concentration was 1 mg/ml, bacteria were eradicated. Additionally, the EG disrupted the structure of the biofilm and killed the embedded bacteria. Importantly, the destruction of membrane integrity and leakage of bacterial cytoplasmic components were induced by the formation of ROS and glutathione reduction. Thus, the EG may provide an efficient strategy to eradicate CRKP and manage related diseases for potential clinical applications.

## Data availability statement

The original contributions presented in the study are included in the article/supplementary material, further inquiries can be directed to the corresponding authors.

## Author contributions

QZ and YS contributed to the conception and design of the study. WL, KD, DW, and GC performed the experiments. WL and GC performed the statistical analysis. WL and KD wrote the first draft of the manuscript. QZ and YS revised the draft of the manuscript and provided the funding. All authors contributed to the article and approved the submitted version.

## Funding

The authors are very grateful for the financial support by National Natural Science Foundation of China (grant no. 31900957, 52103170), Shandong Provincial Natural Science Foundation (grant no. ZR2019QC007), Innovation and technology program for the excellent youth scholars of higher education of Shandong province (grant no. 2019KJE015), Traditional Chinese Medicine Science and Technology Project of Shandong province (grant no. 2021Q069), and Wenzhou Key Laboratory of Biomaterials and Engineering (grant no. WIUCASZZXF21004).

## Conflict of interest

The authors declare that the research was conducted in the absence of any commercial or financial relationships that could be construed as a potential conflict of interest.

## Publisher’s note

All claims expressed in this article are solely those of the authors and do not necessarily represent those of their affiliated organizations, or those of the publisher, the editors and the reviewers. Any product that may be evaluated in this article, or claim that may be made by its manufacturer, is not guaranteed or endorsed by the publisher.
